# Monitoring environmental microbiomes: Alignment of microbiology and computational biology competencies within a culturally integrated curriculum and research framework

**DOI:** 10.1111/1755-0998.13867

**Published:** 2023-09-13

**Authors:** J. S. Lee, J. L. Lowell, K. Whitewater, T. M. Roane, C. S. Miller, A. P. Chan, A. W. Sylvester, D. Jackson, L. E. Hunter

**Affiliations:** ^1^ Department of Chemistry and Biochemistry Fort Lewis College Durango Colorado USA; ^2^ Department of Public Health Fort Lewis College Durango Colorado USA; ^3^ Department of Integrative Biology University of Colorado Denver Denver Colorado USA; ^4^ J. Craig Venter Institute Rockville Maryland USA; ^5^ Marine Biological Laboratory Woods Hole Massachusetts USA; ^6^ University of Wyoming Laramie Wyoming USA; ^7^ Cold Spring Harbor Laboratory Cold Spring Harbor New York USA; ^8^ Department of Biomedical Informatics University of Colorado Anschutz Medical Campus Aurora Colorado USA

**Keywords:** course‐based research experience, cultural integration, data science, environmental microbiology, microbiome

## Abstract

We have developed a flexible undergraduate curriculum that leverages the place‐based research of environmental microbiomes to increase the number of Indigenous researchers in microbiology, data science and scientific computing. Monitoring Environmental Microbiomes (MEM) provides a curriculum and research framework designed to integrate an Indigenous approach when conducting authentic scientific research and to build interest and confidence at the undergraduate level. MEM has been successfully implemented as a short summer workshop to introduce computing practices in microbiome analysis. Based on self‐assessed student knowledge of topics and skills, increased scientific confidence and interest in genomics careers were observed. We propose MEM be incorporated in a scalable course‐based research experience for undergraduate institutions, including tribal colleges and universities, community colleges and other minority serving institutions. This coupled curricular and research framework explicitly considers cultural perspectives, access and equity to train a diverse future workforce that is more informed to engage in microbiome research and to translate microbiome science to benefit community and environmental health.

## 1 INTRODUCTION

Western research methodologies are both approached through the lens of and disproportionally benefit dominant social groups defined by class structure and racialized identity. This has disempowered marginalized communities and systematically excludes community members from science through a lack of informed consent, imposition of stereotypes that reinforce racism and disrespect of cultural and spiritual beliefs and has benefitted individual careers without benefit to the communities struggling with human and environmental health disparities (Garrison et al., 2019; Petereit & Burhansstipanov, 2008). Indigenous communities have been further disempowered through data dependence, a process whereby other entities provide data about tribes, peoples, communities and resources, rather than data sovereignty (Carroll et al., 2019). This phenomenon has led to data collection about Indigenous populations in the United States that are inconsistent and irrelevant to tribal communities and a reliance on data that do not necessarily reflect community needs and priorities. Consequently, meaningful decisions about human and environmental health are extremely difficult and often lead to poor health outcomes (Rainie et al., 2017). Justified distrust of the scientific community has led to a lack of Indigenous community engagement in human genetics research (Garrison et al., 2019). However, it is important that typically underrepresented individuals are included in research so that outcomes are equitable (Fawzy et al., 2022; Petereit & Burhansstipanov, 2008). As genomics and big data become increasingly synonymous with decision‐making, it also becomes increasingly important to train a diverse workforce that can advocate for and act as stewards of data collected by and about marginalized populations.

Recently, there has been a global movement by Indigenous communities from data dependency to data sovereignty (Carroll et al., 2019). By strengthening data collection, interpretation and ownership by and for Indigenous communities, objectives can be better prioritized to secure resources and improve research outcomes. Others have demonstrated that community trust in research can be rebuilt through community‐based participatory research, improving data accuracy and quality, promoting Indigenous methodologies and developing local capabilities (Garrison et al., 2019; Rainie et al., 2017). To achieve these goals, educators must provide workforce development pathways that expose and train diverse students, especially those from Indigenous communities, to meet unique data challenges of the future, while incorporating Indigenous ways of knowing. Training future Indigenous leaders in science, technology, engineering and mathematics (STEM) fields will help strengthen data collection and governance. In turn, research and community advocacy will better reflect values and principles that apply to management and equity of Indigenous nations, thereby increasing health equity and well‐being (Rainie et al., 2017).

Indigenous students represent only 0.5% of college students with the overwhelming majority of bachelor's, master's and doctoral degrees in non‐STEM, service‐oriented disciplines (National Action Council for Minorities in Engineering (NACME), 2012; National Science Foundation, 2018). Indigenous students encounter various barriers, such as inadequate academic preparation, competing family or cultural demands, conflicting epistemologies, income barriers and poor mentorship that prevent them from pursuing or continuing STEM careers (Claw et al., 2018; James et al., 2012). However, Indigenous students were more likely to participate in culturally tailored STEM internships because these programmes provided a sense of belonging to the scientific community that did not conflict with their cultural identities (Chow‐Garcia et al., 2022). This was most likely due to inherently affirming Indigenous perspectives that explicitly explored the relationship between science and Indigenous identity (Page‐Reeves et al., 2019). Indigenous science expresses the interconnectedness of land, water and each other (Bang & Medin, 2010). When Indigenous students feel secure expressing Native/Indigenous and science identities, a sense of belonging within the scientific community can be built, which in turn can increase the number of Indigenous students persisting in science (Chow‐Garcia et al., 2022). To bridge the gap between Indigenous and western science, Begay et al. (2019) suggested that researchers consider cultural perspectives and traditional knowledge resulting in better informed, more ethical and more respectful science.

Course‐based undergraduate research experiences (CUREs) have the potential to engage a much larger number of students in research than traditional individually mentored undergraduate independent study (Corwin et al., 2015; Dolan, 2016) and potentially increase student retention in STEM fields (Fuller & Torres Rivera, 2021; Parks et al., 2020; Rodenbusch et al., 2016; Waynant et al., 2022). The experiential learning environment inherent in CUREs provides a framework in which students can socially interact. The exchange of ideas, backgrounds and shared experiences may increase student participation in their own inquiry‐based learning. Furthermore, the scalability of CUREs to semester‐long or relatively short summer experiences could be especially beneficial at underserved institutions with few resources but that maintain expectations for faculty research. Underserved institutions overlap substantially with minority serving institutions (MSIs) and traditionally include historically Black colleges and universities, Hispanic‐serving institutions, tribal colleges and universities (TCUs), community colleges (CCs) and some primarily undergraduate institutions (PUIs) that support ethnically, racially and socio‐economically marginalized students in the United States (Nguyen et al., 2015; The Genomic Data Science Community Network, 2022). This approach could be especially powerful for removing noninclusive barriers and providing more equitable access to research, thereby improving diversity in the scientific community (Bangera & Brownell, 2014; Estrada et al., 2016).

Significant advances in genomics and microbiome research over the last decade have vastly increased data collection capabilities, requiring the need for broad multidisciplinary computational training (Anton Feenstra et al., 2018; The Genomic Data Science Community Network, 2022) including at the undergraduate level (Shapiro et al., 2013). To meet these needs, best computing practices and common workflows have been developed specifically for microbiome analyses (Shade et al., 2019). Attempts to standardize training in microbiome computational biology have resulted in a set of competencies (Table S1) that highlight common focal points (Mulder et al., 2018; Wilson Sayres et al., 2018). As such, some CUREs have been developed by life sciences faculty to introduce hands‐on bioinformatics training to undergraduate students (Shapiro et al., 2013). For example, an undergraduate 3‐course sequence in microbiome analyses demonstrated beneficial impacts on student bioinformatics competencies (Rosen & Hammrich, 2020). Despite the need for more bioinformatics expertise and the availability of CURE curricula, many barriers prevent the adoption of bioinformatics education and training, especially at MSIs. Most notably, these barriers include a lack of bioinformatics‐trained faculty and limited computing and DNA sequencing resources (Williams et al., 2019). This further emphasizes the need for increasing the accessibility of bioinformatics education and training, especially at MSIs, including those serving Indigenous populations.

Cultural integration in courses can improve and provide a more meaningful educational experience for Indigenous students (Crazy Bull, 2012). The most successful cultural integration is that in which students generate connections between prior life knowledge and course content. Many Indigenous students have a personal relationship and connection to land and community. Obligations to give back to home communities are driving forces for pursuing higher education (Gervais et al., 2017; Johnson et al., 2017). Environmental research inextricably links environmental stewardship and community well‐being (i.e. for Diné Hózhó teachings), thereby increasing the likelihood of engaging Indigenous students in STEM fields (Kahn‐John Diné & Koithan, 2015). Environmental microbiomes, predominantly associated with soils, sediments, rivers, oceans, the built environment and the atmosphere, are foundational to linking humans and the environment (Choudoir & Eggleston, 2022). Microbes impact the world around us, shape large‐scale environmental processes, affect public health and play important roles in industry and biotechnology (U.S. Department of Energy Genomic Science program, 2018). Microbiome research therefore provides a broad yet relevant set of questions on which Indigenous early career researchers can choose to focus while prioritizing tribal and environmental health (Gewin, 2021).

Curriculum in environmental microbiology and microbiome emphasizes the importance of affirming and supporting the need to navigate ethical, cultural and spiritual conflicts (Castagno et al., 2022; Ingram et al., 2021). Research on environmental microbiomes also avoids possible cultural taboos. Examples of cultural and spiritual taboos for some Indigenous people that conflict with STEM practices include the following: (1) archaeological fieldwork of suspected Indigenous burial grounds, (2) surveillance or dissection of specific animals, (3) examination of human cadavers, (4) genetics research, (5) investigation of weather events such as lightning strikes and (5) assigning monetary worth to natural resources (Dorson, 1955; Mathiasen, 2006). Indigenous students are more likely to take science classes if the curriculum is more respectful of cultural taboos (Williams & Shipley, 2018). To support students, the presence of an Elder as a mentor to guide a student and be a positive role model should be considered (Chapman & Whiteford, 2017; Ducharme, 2013). When bringing students from various circumstances, an Elder can provide a cultural space for learning to link back to the culture and values. For example, reminding students to be mindful of the data they are working with and the rationale of the significance to study the connection to land and community.

Following these ethically and culturally guiding principles, and a CURE framework, the Monitoring Environmental Microbiomes (MEM) summer workshop (https://faculty.fortlewis.edu/jslee/MEM/index.html) was designed to incorporate cultural relevance, knowledge and ways of approaching science, utilize many common and widely applicable biochemical techniques and introduce foundational computing skills. The goal of the MEM (previously known as Genomic Science Leadership Initiative, GSLI) workshop was to cultivate a positive learning environment while educating students from diverse backgrounds about genomics, DNA sequencing, molecular biology and computational techniques through environmental microbiome analyses. The workshop design included an environmentally and culturally relevant research project and was an iterative process that spanned several years. Our MEM workshop provides an example of how microbiology and computational sciences can be used to engage more Indigenous students and a framework for further CURE development. Both content delivery options can help address workforce and data literacy needs in tribal communities and at MSIs.

Statement to define Indigenous: Here, we use the term Indigenous, intentionally capitalized, for the political nature of tribal sovereignty. There is no standard descriptor for identifying those who call themselves Native American, American Indian and Alaska Native, or Native. The terms are used interchangeably and seem to be based on preference (Horse, 2005). Indigenous peoples have individual preferences on how they would like to be addressed. Across the United States, federal agencies have historically used American Indian and Alaska Native variations. We acknowledge the variation among the 500+ tribal nations in the United States and respect the differences in their traditions, cultures, languages and worldviews (Grant et al., 2022).

## 2 METHODS

### 2.1 Workshop locations and recruitment

The first genomics workshop began in 2016 and was held at J. Craig Venter Institution in Rockville, MD. This workshop spanned 3 days and focussed on environmental questions related to potential arsenic contamination in local waterways on the Crow Reservation in southern Montana. Using this model, subsequent workshops were held at Fort Lewis College (FLC) in Durango, CO, USA, in 2017, 2018, 2019 and 2022, with an additional location at the University of Colorado Denver (CU Denver), Denver, CO, USA in 2019. Workshop participant selection was prioritized through self‐identification as (1) Indigenous (2) from a marginalized group and (3) a student from a PUI, CC, TCU or MSI. Marginalized groups were defined as: Black/African‐American, Hispanic/Latino(a), Indigenous, Native American/American Indian Alaska Native. A recommendation from a science faculty (or staff) was also required. Workshop hosts networked among PUIs, CCs, TCUs and MSIs to recruit from a wide and diverse applicant demographic. No applicants were excluded based on racial/ethnic background or experience in computational and microbiome skills. Accepted applicants were current undergraduate students majoring in science or science education and/or currently in a science internship with a GPA of at least 2.5. Students were paid a stipend for the week and provided housing and meals. Teaching assistants were recruited from previous workshop years to assist and engage with students.

### 2.2 Relevance of environmental microbiomes, study sites and cultural identity

Microbes are ubiquitous and foundational to life, regulating critical nutrients and elemental cycling (Malla et al., 2018). Microbial communities have great potential for bioremediation in heavily contaminated soil and aquatic environments and the ability to biotransform metals into less toxic forms. Studying topics around microbial roles in the environment and their relevance to ecosystem services perfectly encompasses the interconnectedness of land, water and people, a central tenet to Indigenous science. By centering the workshop on microbiomes in contaminated ecosystems, it was broadly applicable, culturally relevant, and addressed many underlying biological and societally important questions to enhance learning. Two specific study sites were selected for workshops held at FLC (2017–2022) and CU Denver (2019). The first was designed to evaluate water quality and impacts from historic river contamination, and the second was designed to explore the microbiomes of a groundwater bioremediation facility, as detailed below.

#### 2.2.1 Example 1: Acid mine drainage contamination of Cement Creek and the Animas River

There is a long and destructive history of mining in the San Juan Mountains of southwest Colorado. The effects from legacy mining are vast and left upwards of 1500 abandoned mines in its wake (Rodriguez‐Freire et al., 2016). On 5 August 2015, about 3.5 million gallons of acid mine drainage from the Gold King Mine near Silverton, CO, were released into Cement Creek following an accidental breach of an earthen dam (Environmental Protection Agency, 2017). The acid mine drainage, rich in dissolved metals including copper, lead, zinc, arsenic and cadmium, flowed into Cement Creek, a tributary to the Animas and San Juan Rivers, eventually reaching Lake Powell, UT, some 300 miles downstream. The spill affected several communities in its path including Silverton and Durango, CO, Southern Ute Tribal land on the Animas River, and Aztec and Farmington, NM, where the Animas River merges into the San Juan River. Further downstream, the San Juan River borders tribal lands and is considered a sacred river to the Diné (Navajo People). van Horne et al. (2021) studied the impacts of the Gold King Mine spill on the cultural, residential and dietary pathways of the Diné. Dr. Joslynn Lee met with Navajo Nation tribal leaders to discuss and prioritize potential sample collection sites on tribal land. This project became an important topic for the FLC workshops from 2017 to 2022 and allowed for sampling rivers from multiple sites transecting the spill.

#### 2.2.2 Example 2: Groundwater bioremediation facility

The second site was in an urban setting in Denver, CO, with connections to environmental water quality, an area of direct concern for many Indigenous communities. Samples were taken to characterize the microbial community in a successful 1,4‐dioxane bioremediation facility (Cordone et al., 2016). Associated with solvents used in domestic and industrial settings, 1,4‐dioxane has increasingly raised concerns about human and environmental exposures. This allowed students attending the workshop in 2019 at a large urban public university in Denver, CO, to collect samples and ask as‐yet‐unanswered questions about the microbial communities responsible for remediation of contaminants at a local field site.

### 2.3 Microbiome workshop flow

Following our iterative workshop development process, workshops from 2016 to 2018 focussed on wet‐lab techniques, followed by computational skills, while the 2019 workshops incorporated more computational and data science topics alongside the wet‐lab techniques (Table 1a). The 2022 workshop focussed solely on computational and data science topics (Table 1b). All formats used real data sets associated with sample sites for the projects described above. Common focal points regarding microbiome computational biology core competencies were included to keep training in line with undergraduate bioinformatics curricular recommendations (Table S1; Mulder et al., 2018; Wilson Sayres et al., 2018). Accompanying course materials and protocols for each topic are available on GitHub (https://github.com/joslynnlee/qiime2‐workflow‐cyverse/wiki). Because the MEM Workshop was limited to 1 week, water and sediment samples were collected from sample site locations during the month of March preceding the May Workshop. Whole community genomic DNA was extracted and sent to Dr. Noah Fierer's Lab within the Cooperative Institute for Research in Environmental Sciences and the BioFrontiers Sequencing Core at the University of Colorado for 16S rRNA gene amplicon library preparation and Illumina sequencing. Paired‐end Illumina (Miseq V2 2 × 250 reads) sequencing data were provided to students for the data science and microbiome portion of the workshop.

**TABLE 1:** Workshop topics for each focused area.

Day 1	Day 2	Day 3	Day 4	Day 5
(a) Topics per day from workshops run in 2016–2019				
Morning Welcome and Indigenous Blessing Introduction to Overview of Workshop and schedule Off‐campus Field trip to sample sites Introduction to metadata for samples	Lab techniques: (1) DNA extraction and quantitation (2) DNA amplification of 16S rRNA gene (3) Analysis by gel electrophoresis Lecture: Introduction to DNA sequencing	Hands‐on: How to read a journal article Lecture: Introduction to microbiome analysis via QIIME Lecture and Hands‐on: HPC + cloud computing Hands‐on: Introduction to Jupyter notebook Hands‐on: Introduction to UNIX commands via The Carpentries Shell Exercises	Lecture and Hands‐on Learning how to run QIIME 2	Small group project question and work on presentations Presentations Closing Indigenous Blessing
(b) Topics per day from workshop in 2022				
Morning Welcome and Indigenous Blessing Introduction to Overview of Workshop and schedule Off‐campus Field trip to sample sites Introduction to metadata for samples	Hands‐on: How to read a journal article Introduction to microbiome analysis via QIIME Introduction to DNA sequencing Lecture: HPC + cloud computing	Hands‐on: Introduction to UNIX commands via The Carpentries Shell Exercises Hands‐on: Learning how to run QIIME 2 In‐depth Diversity analysis	Data science lecture: Classifiers and Regression Student‐driven classification/regression challenge Hands‐on: Using R to load data and run packages	Small group project question and work on presentations Presentations Closing Indigenous Blessing

Dr. Joslynn Lee is an enrolled member of the Pueblo of Laguna (K'awaika) from her matrilineal side of the family and is of the Diné (Navajo People) from her paternal side of the family. Dr. Lee brings the leadership and lived knowledge to develop a relevant curriculum that encompasses the varying needs of Indigenous students and professionals in STEM. Thus, the workshop schedules consisted of opening remarks and a blessing by our Tribal Elder as part of cultural integration. A field trip to several of the sampling locations gave students the opportunity to visualize the environment from which water and sediment samples were collected, and a sense of how the microbiomes analysed may affect local ecosystems. Successive days included wet‐lab techniques (2016–2019), followed by computational training, small group hypothesis‐driven data analysis and presentations (2016–2022; Table 1).

For the computing portion of the workshop, students were introduced to Jupyter Notebooks (Rule et al., 2019) for Illumina sequencing data analyses combined with detailed data analysis combined with detailed protocols. Within the notebooks, students had the opportunity to iteratively edit, note take and run code within cloud computing environments. Students followed portions of The Carpentries Unix Shell (https://swcarpentry.github.io/shell‐novice/) lessons to learn the basics of file systems and the shell. Students explored the machine learning topic of supervised learning classifiers to predict the categorical metadata classes of unlabelled samples by learning the composition of labelled training samples. Students trained a classifier that predicted the site from which a sample was collected based on microbiome composition. Downstream data analysis enabled students to explore the relationships among sample locations and environmental conditions, for example chemical concentrations, with microbial community structure. For these topics, students learned the statistical software R and more scripting to use plugins from QIIME 2 (https://docs.qiime2.org/2023.5/) (Bolyen et al., 2019). These methodologies are widely applicable and are commonly used in a variety of STEM disciplines. Using Bioconductor with search term ‘microbiome’ yields many bioinformatics software with documentation (https://bioconductor.org/help/search/index.html?q=microbiome/).

### 2.4 Learning assessment

Pre and postworkshop surveys were performed anonymously in Survey Monkey or Google Forms to assess student learning. The survey consisted of 17 questions that were similarly phrased/asked in 2018 and 2019. Learning assessments were focussed solely on these 2 years because questions were consistent among years. Due to workshop content shift in 2022, survey questions differed and could not be directly compared. The student responses in both the pre‐ and postsurveys were rated on a Likert scale 1–5, with 1 meaning that the student demonstrated no knowledge or confidence and 5 meaning the student demonstrated strong knowledge or mastery. Open‐ended questions regarding student attitudes towards the workshop were also included (see Table S2). Participants who submitted only the pre‐ or postsurvey were eliminated. Survey data were deidentified and exported to CSV‐formatted files for analysis and visualization using ggplot2 in R (Wickham, 2016). Survey approval was obtained from the FLC Institutional Review Board (IRB 2022‐75).

## 3 RESULTS

### 3.1 Workshop participation

Workshops started in 2016 and continued through 2019. They were paused in 2020 and 2021 due to the COVID‐19 pandemic. Of the total participants, 82.9% identified as Indigenous, 71.4% as female and 69.9% coming from underserved institutions (PUI/CC/TCUs; Table 2 and Figure S1A–D). Other computational training workshops target marginalized students, but applicants do not always meet the Indigenous demographic (Shade et al., 2019). Building connections with faculty from various schools resulted in students attending from diverse institution types (Table S3). The participants represented over 17 Federally recognized tribes spanning seven US states, with 65% representing the Navajo Nation (AZ, NM and UT; Bureau of Indian Affairs, 2020; Supplementary Material Table S4).

**TABLE 2:** Student participant demographics. Distribution of student ethnicity, gender, academic status and college institution demographics.

	2016		2017		2018		2019		2022		Overall	
	*n* = 10		*n* = 10		*n* = 10		*n* = 30		*n* = 10		*n* = 70	
	*n*	%	*n*	%	*n*	%	*n*	%	*n*	%	*n*	%
Gender												
Male	3	30	4	40	5	50	7	23.3	3	30	22	31.4
**Female**	**7**	**70**	**6**	**60**	**5**	**50**	**23**	**76.7**	**7**	**70**	**48**	**68.6**
Nonbinary	0	0	0	0	0	0	0	0	0	0	0	0
Race/Ethnicity												
**Indigenous** ^a^	**10**	**100**	**9**	**90**	**6**	**60**	**26**	**86.6**	**7**	**70**	**58**	**82.0**
Asian	0	0	0	0	0	0	0	0	0	0	0	0
A/AA/Black^b^	0	0	0	0	0	0	0	0	0	0	0	0
Latino(a)	0	0	1	10	0	0	2	6.7	0	0	3	4.3
White	0	0	0	0	4	4	2	6.7	3	30	9	12.9
Class												
Freshman	2	20	3	30	0	0	6	20	0	0	11	15.7
Sophomore	4	40	2	20	2	2	12	40	1	10	21	30
Junior	2	20	1	10	4	4	8	26.7	5	50	20	28.6
Senior	0	0	4	40	4	4	4	13.3	3	30	15	21.4
Post Bac	2	20	0	0	0	0	0	0	1	10	3	4.3
Institution type												
R1	3	30	0	0	1	10	8	26.7	1	10	13	18.6
R2	2	20	1	10	0	0	4	13.3	0	0	7	10
R3	0	0	0	0	0	0	1	3.3	0	0	1	1.4
**PUI** ^c^	**2**	**2**	**5**	**50**	**5**	**50**	**3**	**10**	**7**	**70**	**22**	**31.4**
**CC** ^d^	**0**	**0**	**0**	**0**	**1**	**10**	**6**	**20**	**1**	**10**	**8**	**11.4**
**TCU** ^e^	**3**	**3**	**4**	**40**	**3**	**30**	**8**	**26.7**	**1**	**10**	**19**	**27.1**

*Note*: Bold Indicates target participants.

Abbreviations: CC, community colleges; PUI; primarily undergraduate institutions; TCU, tribal colleges and universities.

^a^ Indigenous including American Indian and Alaska Native.

^b^ African/African‐American/Black.

^c^ Primarily Undergraduate Institution.

^d^ Community College.

^e^ Tribal Colleges and Universities.

### 3.2 Breadth and exposure to multidisciplinary training

**FIGURE 1:**
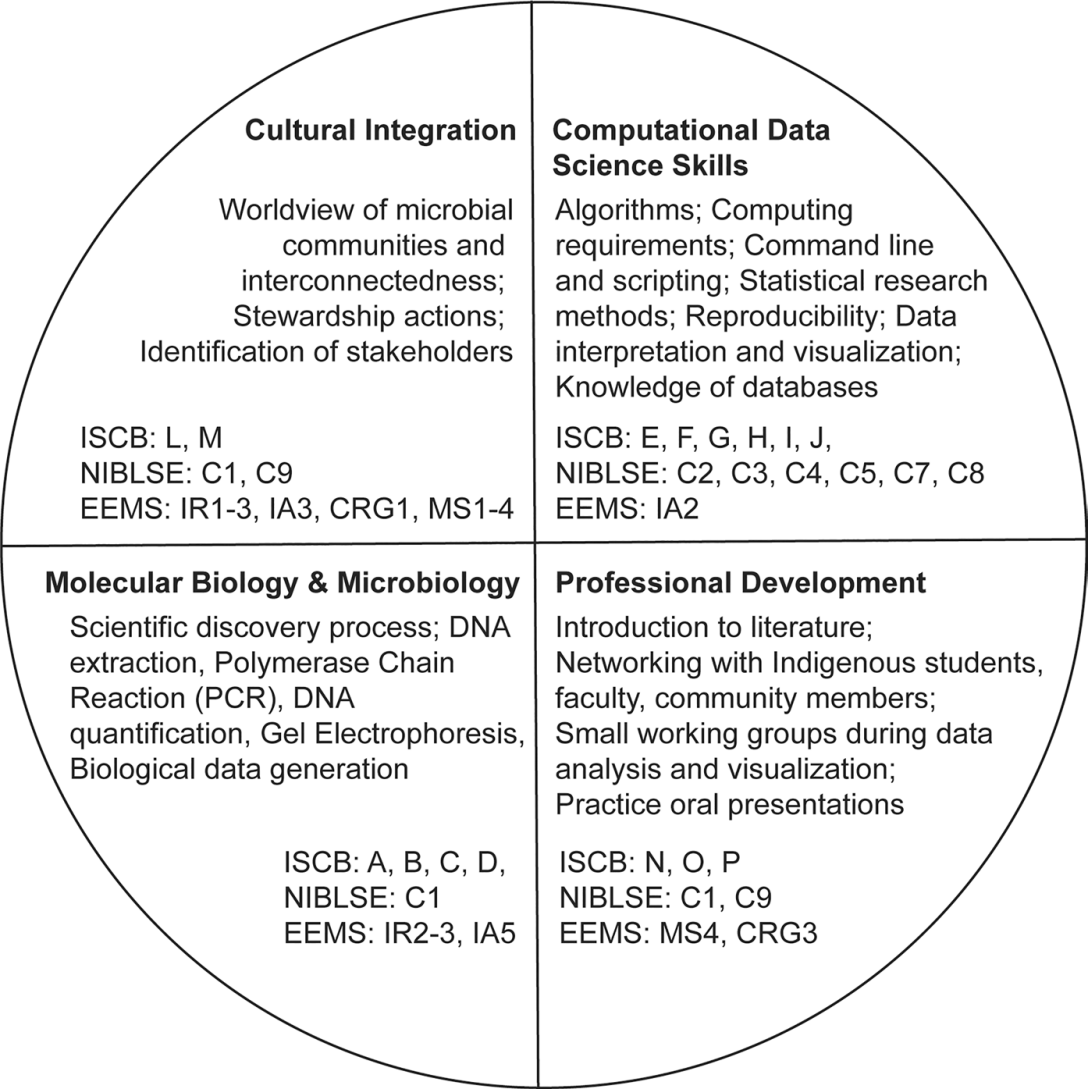
Mapping the skills (NIBLSE), competencies (ISCB) and equitable environmental microbiome stewardship practices (EEMS) into four areas to highlight the topics covered in the workshop.

Students from underserved institutions generally have limited resources and relatively little exposure to genomic data, science curricula and professional development opportunities leading to genomics careers (The Genomic Data Science Community Network, 2022). The MEM workshop sought to teach students bioinformatics core competencies that emphasized utilizing computing resources, data processing (diversity analysis) and data science (downstream processing) applied to environmental microbiomes. There are ‘standard’ bioinformatics tools that have broad applicability across a variety of genomic contexts (Brandies & Hogg, 2021), that is the knowledge base of careers in biomedical research and healthcare (Mulder et al., 2018). Core competencies identified by the International Consortium for Systems Biology (ICSB) curriculum task force (Mulder et al., 2018) and Network for Integrating Bioinformatics into Life Sciences Education (NIBLSE; Wilson Sayres et al., 2018) were combined with guidelines and practices for equitable environmental microbiome research practices. These are targeted at the individual, community and institutional levels (Choudoir & Eggleston, 2022; Figure 1). (See Table S1 for full list of competencies and implementation into MEM workshop).

### 3.3 Learning assessment

**FIGURE 2:**
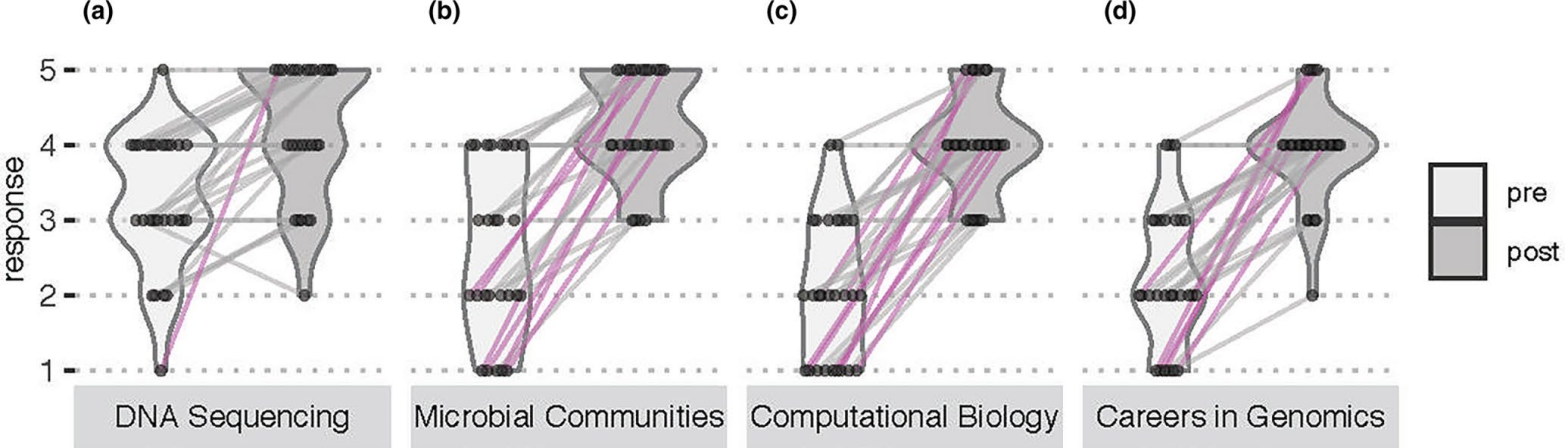
Student survey responses to the question, ‘How much do you currently know about the following topics? Nothing (1), Not so much (2), Neutral (3), A fair amount (4), A lot. (5)’ Preworkshop responses are shown in the left, light grey violin plots, and postworkshop responses in the right, dark grey violin plots for distinct topics (a)–(d). Individual student responses are shown as dots connected by lines, and students with response scores that increased by 3 or more points are highlighted in magenta.

**FIGURE 3:**
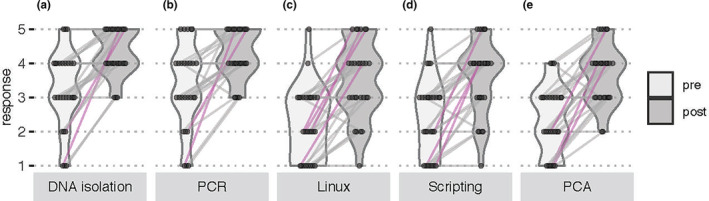
Student survey responses to the question, ‘How confident do you feel about the following lab methods: Isolating DNA from water samples (a), Amplifying DNA by PCR (b), Using the Linux environment and commands (c), Executing a script (d), Generating and examining a Principal Component Analysis plot (e)?’ ‘Not at all confident (1), Not so confident (2), Neutral (3), Confident (4) and Extremely confident (5)’. Preworkshop responses are shown in the left, light grey violin plots, and postworkshop in the right, dark grey violin plots. Individual student responses are shown as dots connected by lines, and students with response scores that increased by 3 or more points are highlighted in magenta.

Of the 40 participants in the 2018–2019 workshops, 27 completed (67.5% response rate) both pre and postworkshop surveys. Paired data responses assessing knowledge and confidence of laboratory and computational methods suggested a self‐assessed shift from little knowledge and confidence to ‘a lot of knowledge’ and ‘confident’ and ‘extremely confident’ in most workshop activities (Figures 2 and 3). The most significant gains were in knowledge of microbial communities, computational biology and genomics careers, with the majority of survey respondents reporting an increase from two to four or five units on a five‐unit Likert scale (Figure 2b–d). This suggested that we met our workshop goals of exposing participants to the importance of environmental microbiome analyses and careers within microbiology, data science and scientific computing.

**FIGURE 4:**
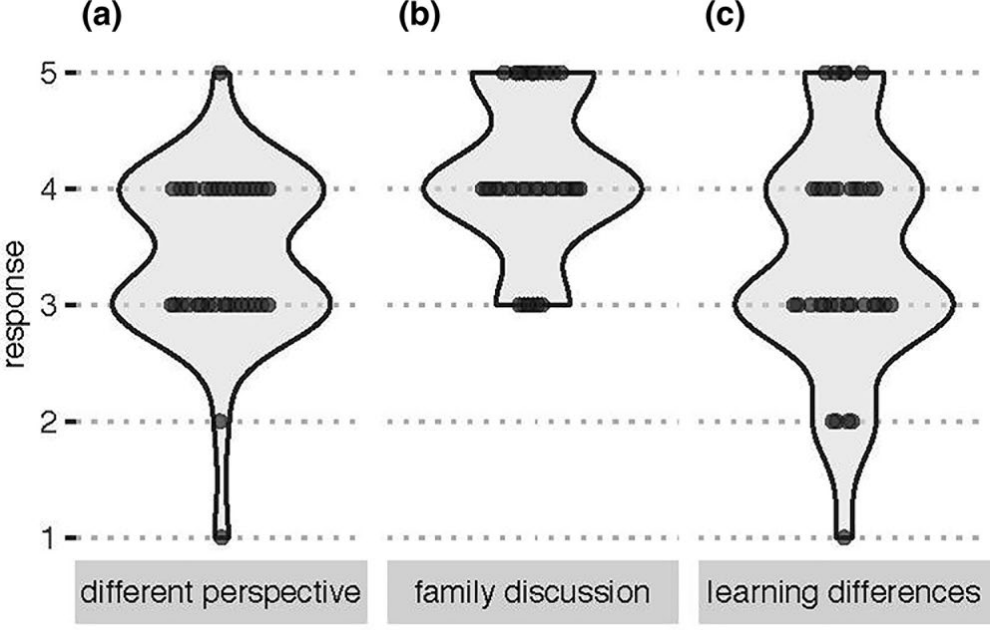
Student survey responses about perceptions of western science versus Indigenous ways of thinking. Students were asked to respond to the statements, ‘I have a different perspective on western science than my non‐Native American/American Indian peers at school’ (a), ‘I am comfortable discussing western science with my family’ (b), and ‘I learn western science differently than non‐Native American/American Indian students because of my Native American/American Indian cultural background’ (c). Responses were Strongly disagree (1), Disagree (2), Neither agree nor disagree (3), Agree (4) and Strongly agree (5).

An additional workshop goal was to explore how students perceived cultural identities in STEM. The questions regarding perceptions of western science versus Indigenous ways of thinking were asked to understand whether creating a place‐based culturally responsive research question is needed for training. Although the postsurvey did not include the same questions, preworkshop surveys highlighted that many participants agreed they learn western science differently than their Non‐Native American/American Indian peers (Figure 4). Gaining this insight was incredibly useful for guiding curricula and agreed with what others have previously reported (Brayboy & Castagno, 2008). When asked ‘What have you liked best about your trip as a whole?’, students overwhelmingly reported their enjoyment of and importance in being engaged with a learning community, especially with other Indigenous scientists (Table 3). Open‐ended question responses suggested that including cultural perspectives and traditional knowledge within our workshop helped students feel included in the broader STEM community and helped bridge the gap between Indigenous and western science. The experiential learning environment provided within the MEM workshops allowed various positive social interactions (i.e. peer‐to‐peer, student‐to‐Tribal Elder and student‐to‐instructor), exchange of ideas, backgrounds and shared experiences that helped students fully engage in workshop content.

**TABLE 3:** Representative statements from anonymous survey. The MEM workshop was previously called Genomic Science Leadership Initiative (GSLI), which is referenced in the statements below.

**Comment content regarding open‐ended responses to the question: ‘What have you liked best about your trip as a whole?’**
Analysing data with QIIME 2, learning about Jupyter notebooks, discussions on Indigenous science and connections between this and Western science
Working with professionals in the field and meeting other Native students studying science
Learning a new skill with other people and everyone being excited about science
Learning about new and efficient computational methods for microbial data analysis, learning from Indigenous scholars and scientists and learning with future Indigenous scholars and scientists
I liked how I could be a part of a community learning about science. With people who have science backgrounds or want to go into science. Also, us being all Native American made this workshop feel more comfortable.
The passion for Indigenous and science and all the wonderful and intelligent faculty that arranged the workshop and helped throughout the week.
Spending time with Native scholars because they are all unique and people I now consider part of the GSLI family for life.
I loved everything! It was filled with so much knowledge that I wish we had more time to spent on each subject! It felt amazing to get a taste of what its like to conduct a research process. Thank you for the experience!
I liked creating a community and learning lots of new information I didn't even know I needed.
I really liked meeting other Native American students who also have an interest in the field of molecular biology and genomics. For me, it was a different experience to have lab and lectures filled with Native American students, I felt more comfortable learning the new material. I also really liked the instructors who helped the students throughout the workshop, it was a great way to make connections.
Loved getting to meet new people from various parts of the country and their unique backgrounds

## 4 DISCUSSION

### 4.1 Workshop format and science topic considerations

The workshops were held in late May, which created an additional summer research opportunity for both faculty and students. In early 2016 and 2017, the workshops were 3 days. One of the important lessons learned was directly engaging with local tribal faculty and Elders and integrating their participation to provide support and guidance to the students and the workshop (Kahn‐John Diné & Koithan, 2015). In addition to being aware of recruitment and building the relationships across institutions of power dynamics, the use of the collective impact model (Kania & Kramer, 2011) was important for the success of this workshop and training. Survey responses from participants requested to extend the workshop to 5 days due to the amount of content. In 2018, the workshop included more time particularly devoted to computational methods. With funding changes in 2019, the workshop focussed more on data science concepts. Instead of a focus on lecture‐driven instruction, the sequence of topics in the workshop allowed students an introduction to topics followed by extensive active practice of application of skills to a meaningful real‐world research data set. The curriculum can be modified or adapted for any student audience (e.g. major, nonmajor and expert or beginner.)

### 4.2 Building a positive learning community and network

Many of the survey responses included statements reflecting a safe and inspirational space knowing other Native American/Indigenous students. Statements in Table 3 highlight the importance of the unique nature of selecting a higher number of Indigenous students in the cohort. Building an inclusive Indigenous curriculum in microbiome research required culturally responsive teaching, which can begin with (1) integrating worldviews and epistemologies of students into the curriculum, (2) using oral stories and elders in the classroom and (3) adapting the classroom to look and feel more like the local community (Brayboy & Castagno, 2008). Building connections with students shows them that science can be relevant to their lives, helps them be engaged and become eager to learn, resulting in a more positive attitude towards science (Brayboy & Castagno, 2008). A vast majority of educators lack the necessary knowledge to provide an effective, high‐quality and culturally responsive education (Belgarde et al., 2002); however, the MEM structure and curriculum provides a framework for educators to integrate these cultural perspectives into courses. This workshop served Indigenous/American Indian and Alaska Native students and acknowledged this student population served.

Of note was starting and ending the workshops with an Indigenous blessing by the Tribal Elder‐in‐residence, who introduced an intentional mindset for all participants. Furthermore, the example of Hózhó teachings was highlighted in the introduction to share that Diné have high regard for animals, living creatures, nature and elements of the earth because of their influence (both negative and positive) on the health and well‐being of human beings. This reflective space generated Indigenous ways of learning and thinking, which was commented on by students as making the experience more holistic.

A classroom climate that builds relationships between teachers and students and between students fosters positive relationships that can impact student exploration of new learning opportunities or allow them to approach teachers easily, increasing academic performances (Hughes & Kwok, 2007; Pianta, 1999). To build upon peer‐to‐peer mentoring, 2–3 participants from previous years were invited back as teaching assistants (TAs; not included in participant data Table 1). Increases in confidence and knowledge levels (Figures 2 and 3) could be tied to TAs creating a safe learning environment because the TAs were students themselves. Workshop participants were likely to be comfortable making mistakes in a low‐stakes environment. Students were not given course credit but were compensated for participating in the course. During the workshop, we encouraged students to support one another while troubleshooting, also highlighting the peer‐to‐peer interactions. The workshop allowed participants from various states and institutions to interact, especially students from various tribal nations.

### 4.3 Continued research opportunities

Many students who participated in the workshops continued in STEM research or professional school, and various faculty involved as instructors and mentors received indirect benefits from this experience. (Table 4) Dr. Larry Hunter had one student join the PhD in biosciences graduate programme in 2022 and another student joining a biosciences summer internship immediately following the 2019 workshop. One faculty member from Northwest Indian College attended the workshop in 2019 and her student published sequencing work (Hart et al., 2022). Drs. Joslynn Lee and Jennifer Lowell have mentored various students from the 2019 and 2022 workshops who continued during the academic year with microbiome research projects for their senior theses. Other noteworthy outcomes (Table 4) include: three students entered PhD biomedical programmes, one in a MS programme in microbiology, three attending Medical School, two in professional programmes (DMD/Vet) and one high school STEM teacher on the Navajo Nation reservation. Not all students were tracked postworkshop, so these outcomes represent a minimum estimate of the impact on student retention/continuation in STEM.

**TABLE 4:** Long‐term outcomes of students per year in various programmes and professions.

Types of programmes/Profession	Year students attended			
	2016	2017	2018	2019
PhD Biomedical programme	1	1		1
MS Microbiology programme			1	
Medical school				3
Professional Programs (DMD/Veterinary school)			2	
High school STEM teacher		1		

## 5 CONCLUSION—FUTURE DIRECTIONS

Indigenous students face disproportionate barriers to mentored independent undergraduate research in part because of the inequitable (or exclusive) approaches used by higher education institutions that frequently serve this population. Others have highlighted the barriers present to obtaining so‐called ‘apprentice‐based research experiences’ in general, often because of the faculty‐to‐student ratio. This is true at many tribal colleges and community colleges. An alternative model is to use centralized, cross‐institutional infrastructure and curriculum, which facilitates scalable CUREs where students and faculty share discovery and resources across multiple campuses, lowering the burden for access to research experiences not only for students but also for faculty and institutions.

We envision that the place‐based research framework we have developed in a workshop format could easily be extended to semester‐long CUREs at a network of partner institutions, which would provide many students with access to research experiences. Importantly, these experiences could be offered at tribal colleges and other settings with fewer expectations and resources for faculty research. This model requires initial up‐front costs for instructor training, but then benefits from reduced costs associated with cross‐institutional sharing of instructor mentorship, access to research infrastructure (such as shared high‐throughput sequencing runs) and shared resources for student and faculty dissemination of research. As a model, the SEA‐PHAGES and SEA‐GENES projects have leveraged this with partnership with New England Biolabs and Integrated DNA Technologies to build an arrayed genomic plasmid library (Heller & Sivanathan, 2022). In SEA‐PHAGES, several self‐assessed metrics of intent to persist in science (such as science identity and science community value) were higher for students participating in the CURE than students in traditional laboratory courses (Hanauer et al., 2017). Importantly, these gains in persistence were equally strong for first‐generation college students and students from marginalized communities in STEM fields and students regardless of institution type, a result replicated elsewhere (Rodenbusch et al., 2016). The Genomics Education Partnership (https://thegep.org/) provides training and resources for faculty implementing CUREs and provides community. We have already begun adapting the curriculum and research framework developed for MEM for use in introductory General Biology laboratories and hope in the near future to develop a network of place‐based environmental microbiome research that benefits multiple institutions.

## AUTHOR CONTRIBUTIONS

JSL designed the cultural integration and computational sequence of project and wrote most of the manuscript. AWS, APC and DJ conceived and designed the wet‐lab sequence. AWS, APC, CM DJ, JLL, JSL, LH and TR offered and taught the workshops. CM and JLL conducted and summarized data analysis. JSL and KW generated the computational workflow for workshop materials. All authors have been involved in drafting the paper or revising it critically, give final approval of the submitted version and agree to be accountable for all aspects of the work.

## FUNDING INFORMATION

The material and 2016–2018 workshop funding were supported by the National Science Foundation (grant number 1027445) to DJ, AWS and APC. The material and 2019 workshop funding were supported by the NIH National Library of Medicine training grant (grant number T15LM009451‐12S1) and the committed funding from the University of Colorado Cancer Center grant to LH.

## CONFLICT OF INTEREST STATEMENT

The authors declare that the research was conducted in the absence of any commercial or financial relationships that could be construed as a potential conflict of interest.

## BENEFIT‐SHARING STATEMENT

Benefits Generated: Dr. Joslynn Lee met with Navajo Nation tribal leaders to discuss and prioritize potential sample collection sites on tribal land for the *Acid Mine Drainage Contamination of Cement Creek and the Animas River project*. Dr. Chris Miller and Dr. Timberley Roane met with managers of the local bioremediation facility to discuss sampling for the *Groundwater Bioremediation Facility project*. Samples collected were used to generate genomic data used by workshop participants to produce presentations to summarize important findings. Short‐term summaries were shared with relevant communities, but long‐term summaries are in preparation. All deidentified survey data and analysis has been uploaded to GitHub. The workflows to analyse genomic data used by workshop participants have been uploaded to GitHub.

## ETHICS STATEMENT

The study was reviewed and approved by the Fort Lewis College Institutional Review Board (IRB 2022‐75).

## Supporting information


Data S1:

## Data Availability

The source code and survey data for the analysis have been made available from GitHub (https://github.com/joslynnlee/MEMSurveys, doi: 10.5281/zenodo.8256953). The workshop material developed, and tutorials have been made available from GitHub (https://github.com/joslynnlee/qiime2‐workflow‐cyverse/wiki, doi: 10.5281/zenodo.8256996).
