# Supplementary material for: Monitoring environmental microbiomes: Alignment of microbiology and computational biology competencies within a culturally integrated curriculum and research framework
Source: Mol Ecol Resour. 2023 Sep 13;25(2):e13867. doi: 10.1111/1755-0998.13867 (PMC11696487; doi:10.1111/1755-0998.13867)

**Supplemental Information for:**

**Monitoring Environmental Microbiomes (MEM): Alignment of Microbiology and Computational Biology Competencies within a Culturally Integrated Curriculum and Research Framework**

Lee, JS, Lowell, JL, Whitewater, K, Roane, TM, Miller, CS, Chan, AP, Sylvester, AW, Jackson, D, Hunter, LE

**Table of Contents:**

| **Table S1. Core Competencies ICSB, NIBLSE, and Environmental Microbiome Research Practices (EEMS)** | Page 2 |
| --- | --- |
| **Table S2. Survey questions from 2018 and 2019** | Page 9 |
| **Table S3. List of Institutions from students provided information** | Page 10 |
| **Table S4. List of Federally Recognized Tribes students provided information** | Page 11 |
| **Supplementary Figure 1** | Page 12 |

Table S1. Core Competencies ICSB, NIBLSE, and Environmental Microbiome Research Practices (EEMS)

| **Label** | **Skills, Competencies and Practices** | **Integration in MEM workshop** |
| --- | --- | --- |
| **Section 1. Core competencies A through P from “The development and application of bioinformatics core competencies to improve bioinformatics training and education”:** <https://journals.plos.org/ploscompbiol/article?id=10.1371/journal.pcbi.1005772> | | |
| A | General Biology | Understanding of central dogma of molecular biology, evolution/phylogeny, and cellular structure. |
| B | Depth in at least one area of biology (e.g. evolutionary biology, genetics, molecular biology, biochemistry, anatomy, physiology) | Knowledge and application of molecular biology, including key techniques: DNA extraction, Polymerase Chain Reaction (PCR), DNA quantification, and Gel Electrophoresis. |
| C | Biological data generation technologies | Knowledge and application of high-throughput sequencing methods. |
| D | Details of the scientific discovery process and of the role of bioinformatics in it | Design a research question to learn about planning a microbiome study. |
| E | Statistical research methods in the context of molecular biology, genomics, medical and population genetics research | Application of statistical methods to evaluate community diversity. |
| F | Bioinformatics tools and their usage | Application of microbiome bioinformatics software QIIME 2 and R packages for analyzing and visualizing data. |
| G | The ability of a computer-based system, process, algorithm, component, or program to meet desired needs in scientific environments/problem | Knowledge of access to computing systems to run programs on large biological datasets. |
| H | Computing requirements appropriate to solve a given scientific problem (e.g. system, process, algorithm, component or program; define algorithmic time and space complexities and hardware resources required to solve a problem | Application to connect to high performance computing resources (Amazon Web Services or CyVerse). |
| I | GUI/Web-based computing skills appropriate to the discipline (e.g. effectively use bioinformatics and analysis tools through web) | Comprehension and application of web-based tools to use databases and for data interpretation. |
| J | Command line and scripting based computing skills appropriate to the discipline | Application and comprehension of Linux-based systems. Applied The Carpentries Unix Shell lessons to navigate remote/local file systems and execute scripts for bioinformatics tools. |
| K | Construction of software systems of varying complexity based on design and development principles | Did not apply |
| L | Local and global impact of bioinformatics and genomics on individuals, organizations, and society | Comprehension on the scale of computing to answer workshop research questions and goals. |
| M | Professional, ethical, legal, security, and social issues and responsibilities of bioinformatics and genomics data in the workplace | Understanding how to use the real data sets and relate the results to impacts of public health. |
| N | Effective communication of bioinformatics and genomics problems/issues/topics with a range of audiences, including, but not limited to, other bioinformatics professionals. | Comprehension of topics and techniques to apply with real data sets to answer workshop research goals. |
| O | Effective teamwork to accomplish a common scientific goal | Evaluating real data sets to answer workshop research goals. |
| P | Engage in continuing professional development in bioinformatics | Meeting with diverse scientists, researchers, educators, and peers within the field. |
| **Section 2. Core competencies C1 through C9 from “Bioinformatics core competencies for undergraduate life sciences education”:**<https://journals.plos.org/plosone/article?id=10.1371/journal.pone.0196878> | | |
| C1 | Explain the role of computation and data mining in addressing hypothesis-driven and hypothesis-generating questions within the life sciences. | Understanding the role of 16S rRNA databases in classification. Generating hypotheses about microbiome associations with site chemistry. Application of classifiers to large 16S datasets. |
| C2 | Summarize key computational concepts, such as algorithms and relational databases, and their applications in the life sciences | Knowledge of access to computing systems to run programs on large biological datasets. |
| C3 | Apply statistical concepts used in bioinformatics | Application of statistical methods to evaluate community diversity. |
| C4 | Use bioinformatics tools to examine complex biological problems in evolution, information flow, and other important areas of biology | Apply bioinformatics tools and “tree thinking” for generating, visualizing, and interpreting 16S phylogenies. |
| C5 | Find, retrieve, and organize various types of biological data | Comprehension and application of web-based tools to use databases and for data interpretation. |
| C6 | Explore and/or model biological interactions, networks, and data integration using bioinformatics | Did not apply |
| C7 | Use command-line bioinformatics tools and write simple computer scripts | Application and comprehension of Linux-based systems. Application of microbiome bioinformatics software QIIME 2 and R packages for analyzing and visualizing data. Application of Jupyter notebooks and cloud-based computing environments. |
| C8 | Describe and manage biological data types, structure, and reproducibility | Understand high-throughput sequencing data types, including quality scores. |
| C9 | Interpret the ethical, legal, medical, and social implications of biological data | Integrated throughout place-based, culturally responsive workshop design. |
| **Section 3: Action Levels From the “Reciprocal Inclusion of Microbiomes and Environmental Justice Contributes Solutions to Global Environmental Health Challenges”:**<https://journals.asm.org/doi/full/10.1128/msystems.01462-21> | | |
| Individual reflection  (IR) 1 | Re-examine your own thinking around the connections between microbes and environmental, animal, human, and plant health. Recalibrate your relationship to microbes. | Bridge discussions around environmental justice and the importance of microbes within projects. |
| Individual reflection  (IR) 2 | Reframe our thinking around biological connections, from molecular to microbial to planetary scales | Project topics discuss and evaluate the connections between microbial communities and public health concerns of contaminated water systems. |
| Individual reflection  (IR) 3 | Critically reflect on who has the expertise and who is considered expert. Challenge the notions of what makes someone a scientist, or an environmental justice advocate, and uplift these individuals or communities. | Elder-in-residence are invited to share their cultural and environmental knowledge. |
| Individual action  (IA) 1 | Create and contribute to antiracist STEM communities (research groups, programs, departments, and beyond). | Built into the learning environment and experience of the workshop. |
| Individual action  (IA) 2 | Actively build anti-oppressive academic research groups. Set goals to build inclusivity and equity into microbiome research, collaborations, and training | Built into the learning environment and experience of the workshop. |
| Individual action  (IA) 3 | Contribute your expertise to social or environmental justice activism. How can you inform local, regional, national, or global scale policy change? With whom might you partner to advocate for and implement these changes? | Students learn about the engagement of local communities affected by water quality issues. |
| Individual action  (IA) 4 | Exercise equitable citational practices. Who are you citing in your manuscripts? Gender balance? Are you citing BIPOC scholars? How can you cite Indigenous knowledge? | Not actively applied in the workshop. |
| Individual action  (IA) 5 | Prioritize physical and mental safety when conducting field work for yourself, your colleagues, and any students or trainees with whom you work. Engage in inclusive, accessible, and safe field work. | Students and faculty external to workshop participants adhere to cultural and accessible field work. |
| Community & research group (CRG) 1 | Move beyond ethical research guidelines and towards dismantling colonial legacies in research institutions, projects, and within ourselves as scholars | Built into the learning environment and experience of the workshop. |
| Community & research group (CRG) 2 | Support, collaborate with, and hire BIPOC scholars. Invite BIPOC scholars to meetings and talks to share their research expertise. Representation matters in conference, departmental, keynote, panelist, and seminar speaker series. | Built into the learning environment and experience of the workshop. |
| Community & research group (CRG) 3 | Practice ethical publication. Where are you publishing? Is your research open access? Are your data and code available and reproducible? How are you sharing your research with diverse audiences and stakeholders? Information access in environmental microbial ecology is still predominantly academic and privileged. Commit to basic, applied, and translational research that addresses power structures and broadens the scope and standards of our scholarship communication. This is especially important in communities historically excluded from this work. | Long-term sample collection and measurement mindset is taking place along the workshop in a research scope. The goal of the Fort Lewis College workshop is to engage with diverse stakeholders, especially those from Navajo Nation. |
| Microbiome stewardship (MS) 1 | Consider how your personal, professional, and recreational activities alter microbial ecosystems. | Students learn about the engagement of local communities affected by water quality issues. |
| Microbiome stewardship (MS) 2 | How do your individual and collective consumer behaviors relate to environmental microbiomes within built and managed environments? To the public health of your local communities? | Students learn about the engagement of local communities affected by water quality issues. |
| Microbiome stewardship (MS) 3 | Engage in community-based research. Accept that there is a different timeline for this. Advocate for institutional and structural changes that prioritize engagement with and funding of this work. | Long-term sample collection and measurement mindset is taking place along the workshop in a research scope. The goal of the Fort Lewis College workshop is to engage with diverse stakeholders, especially those from Navajo Nation and local communities. |
| Microbiome stewardship (MS) 4 | Build relationships with diverse stakeholders to more effectively generate questions, and to promote research that meaningfully engages with local and regional communities. | Students comprehend the diverse stakeholders as they prepare presentations to disseminate information. |
| Microbiome stewardship (MS) 5 | Translating principles of community-based participatory research across disciplines offers guidance for research carried out with communities to effect change in environmental microbiome research. | Long-term sample collection and measurement mindset is taking place along the workshop in a research scope. The goal of the Fort Lewis College workshop is to engage with diverse stakeholders, especially those from Navajo Nation and local communities. |

Table S2. Survey questions from 2018 and 2019

| **Q1 - Q5:** How much do you currently know about the following (5) topics?  Nothing (1), Not so much (2), Neutral (3), A fair amount (4), A lot (5)  **Pre- and post-workshop** |
| --- |
| Q1. DNA sequencing |
| Q2. Polymerase Chain Reaction (PCR) |
| Q3. Microbial communities |
| Q4. Computational biology |
| Q5. Careers in genomics |
| **Q6-10:** How confident do you feel about the following (5) lab methods?  Not at all confident (1), Not so confident (2), Neutral (3), Confident (4), Extremely confident (5)  **Pre- and post-workshop** |
| Q6. Isolating DNA from water samples |
| Q7. Amplifying DNA by PCR |
| Q8. Using the Linux environment and commands |
| Q9. Executing a script |
| Q10. Examining a Principle Component Analysis plot |
| **Q11-13:** For the following statements, please indicate your level of agreement using the scale below: Strongly disagree (1), Disagree (2), Neither agree nor disagree (3), Agree (4), Strongly agree (5) **Only Pre-workshop** |
| **Q11.** I have a different perspective on western science than my non-Native American/American Indian peers at school. |
| **Q12.** I am comfortable discussing western science with my family. |
| **Q13.** I learn western science differently than non-Native American/American Indian students because of my Native American/American Indian cultural background. |
| **Q14:** How do you define genomics? (open-ended response)  **Pre- and post-workshop** |
| **Q15:** Please provide any suggestions you have to make this a better workshop: (open-ended response)  **Post-workshop** |
| **Q16:** Fill in the blank: On this trip, I ____________________ for the first time. (open-ended response) **Post-workshop** |
| **Q17:** What have you liked best about your trip as a whole? (open-ended response)  **Post-workshop** |

Table S3. List of Institutions from students provided information

| **Institution** | **2016** | **2017** | **2018** | **2019** | **2022** | **Total** |
| --- | --- | --- | --- | --- | --- | --- |
| Blackfeet Community College |  |  |  | 2 |  | 2 |
| Central NM Community College |  |  |  | 1 |  | 1 |
| Coconino Community College |  |  |  |  | 1 | 1 |
| Colorado State University |  |  |  | 1 |  | 1 |
| University of Colorado Denver |  |  | 1 | 2 | 1 | 4 |
| Diné College |  | 4 | 3 | 2 |  | 9 |
| Fort Lewis College | 2 | 5 | 5 | 3 | 7 | 22 |
| Little Bighorn College | 2 |  |  |  |  | 2 |
| Montana State University | 4 |  |  |  |  | 4 |
| Northern Arizona University | 2 | 1 |  | 4 |  | 7 |
| Northwest Indian College |  |  |  | 4 | 1 | 5 |
| Pacific College |  |  |  | 1 |  | 1 |
| Phoenix College |  |  |  | 2 |  | 2 |
| San Juan College |  |  | 1 | 2 |  | 3 |
| University of Arizona |  |  |  | 5 |  | 5 |
| Utah State University Eastern Blanding |  |  |  | 1 |  | 1 |
| **Total** | **10** | **10** | **10** | **30** | **10** | **70** |

Table S4. List of Federally Recognized Tribes students provided information

| **Federally Recognized Tribes**  [**https://www.ncsl.org/legislators-staff/legislators/quad-caucus/list-of-federal-and-state-recognized-tribes.aspx**](https://www.ncsl.org/legislators-staff/legislators/quad-caucus/list-of-federal-and-state-recognized-tribes.aspx) | **Total** |
| --- | --- |
| Blackfeet Tribe of the Blackfeet Indian Reservation of Montana | 2 |
| Chippewa-Cree Indians of the Rocky Boy's Reservation | 1 |
| Confederated Tribes of the Colville Reservation | 1 |
| Crow Tribe of Montana | 2 |
| Fort Belknap Indian Community of the Fort Belknap Reservation of Montana | 1 |
| Hopi Tribe of Arizona | 1 |
| Kiowa Indian Tribe of Oklahoma | 1 |
| Lummi Tribe of the Lummi Reservation | 2 |
| Muscogee (Creek) Nation | 1 |
| Navajo Nation (Arizona, New Mexico and Utah) | 38 |
| Nooksack Indian Tribe of Washington | 1 |
| Northern Cheyenne Tribe | 1 |
| Oglala Sioux Tribe | 2 |
| Confederated Salish and Kootenai Tribes of the Flathead Reservation | 1 |
| Kewa Pueblo | 1 |
| Tulalip Tribes of Washington | 1 |
| White Mountain Apache Tribe of the Fort Apache Reservation | 1 |
|  | 58 |

**Supplementary Figure 1.** Student participant demographics. A) Percent participation by race/ethnicity. B) Percent participation by academic status. C) Percent participation by gender. D) Percent participation by institution type.

#
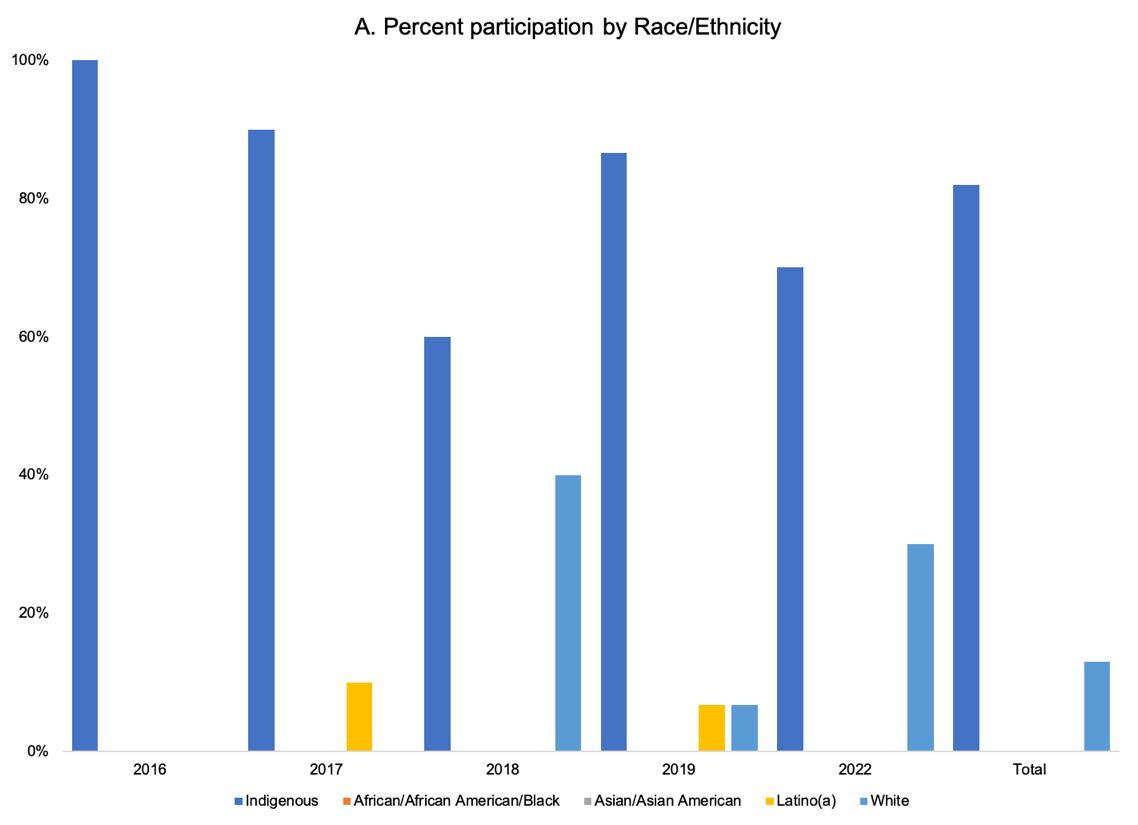


#
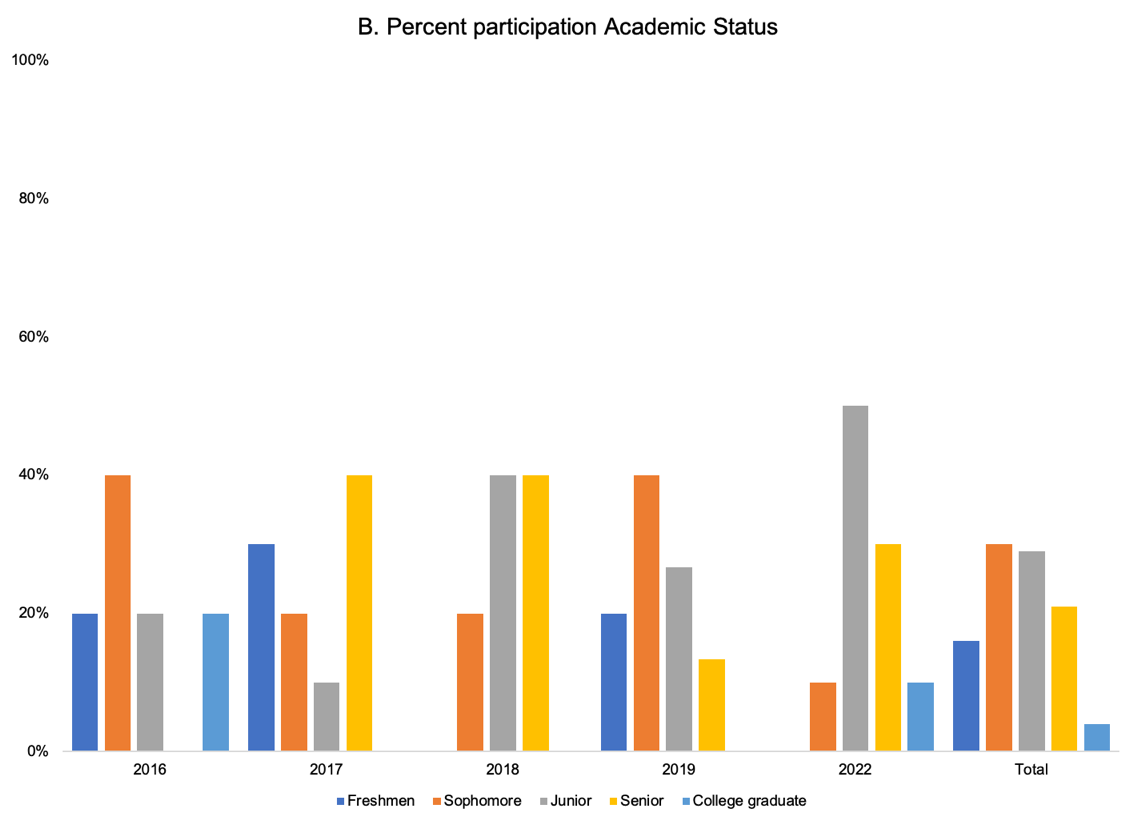


#
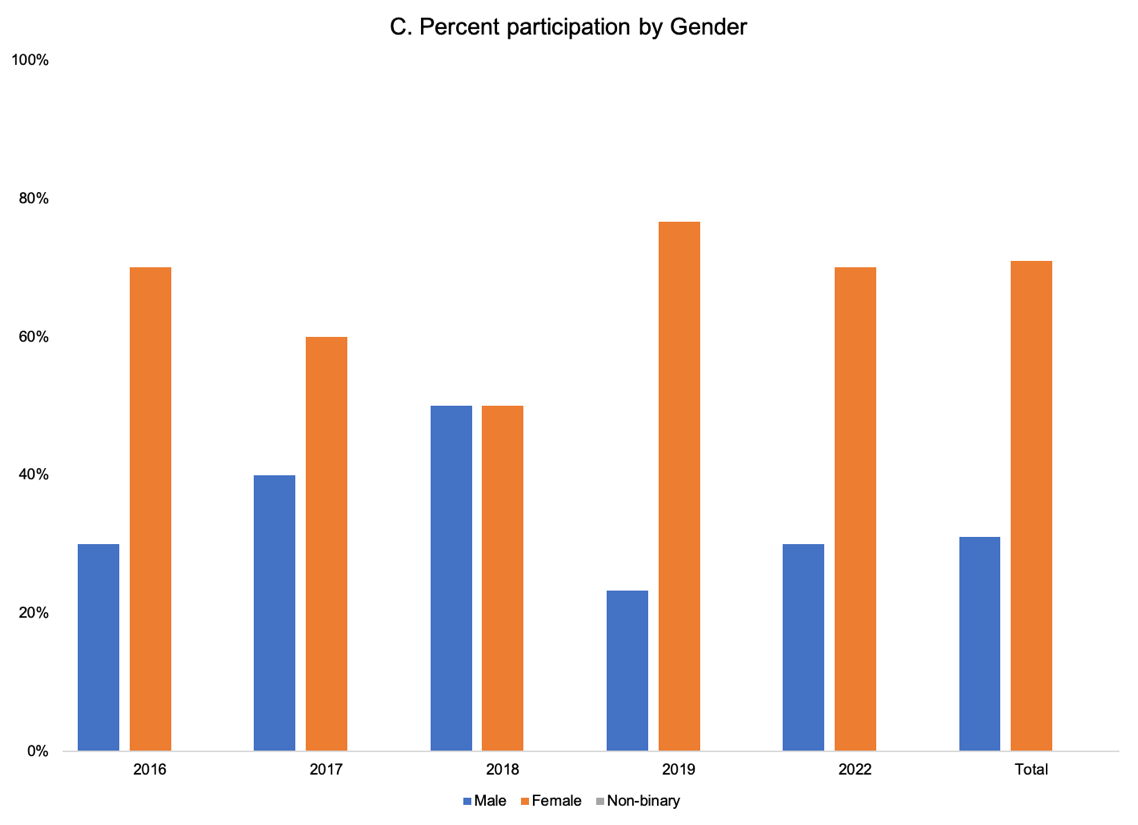


#
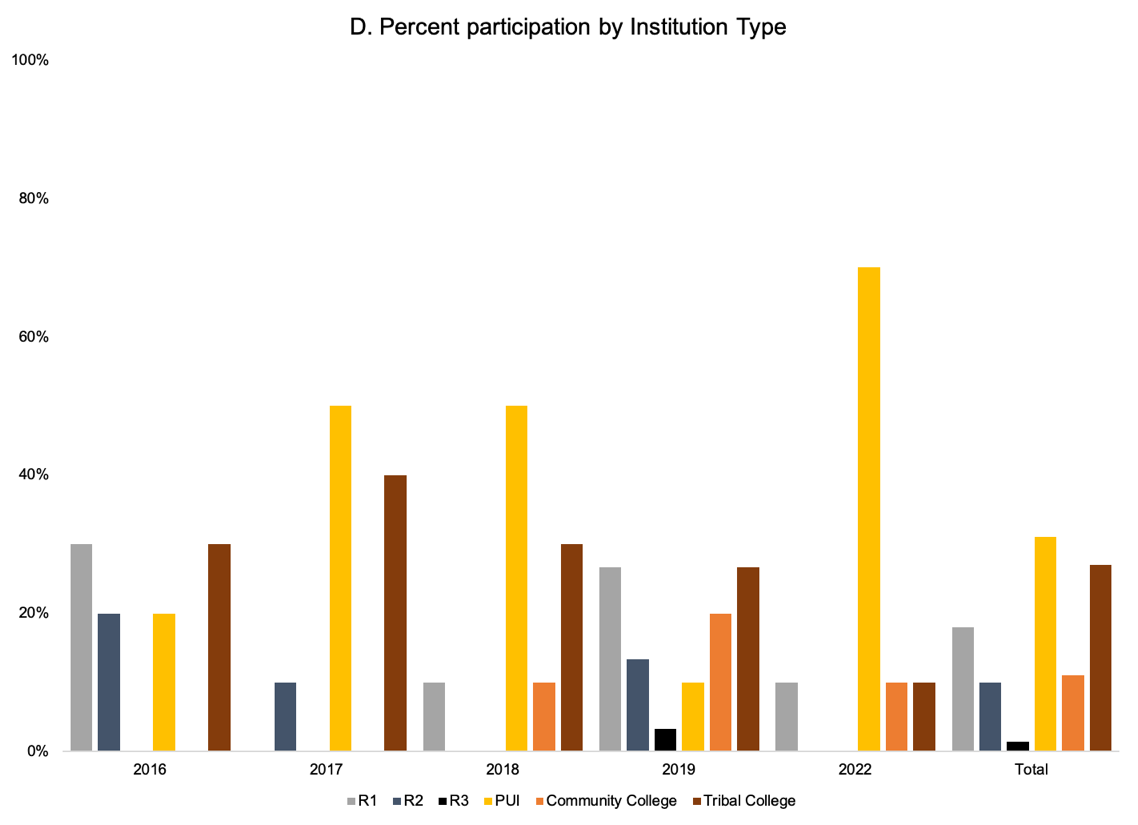

Supplement: Supplementary file 1 — Data S1: [file MEN-25-e13867-s001.docx]
